# Supplementary material for: Lexical phylogenetics of the Tupí-Guaraní family: Language, archaeology, and the problem of chronology
Source: PLoS One. 2023 Jun 15;18(6):e0272226. doi: 10.1371/journal.pone.0272226 (PMC10270611; doi:10.1371/journal.pone.0272226)
Supplement: S1 File — (PDF) [file pone.0272226.s001.pdf]

Appendix A Concepts used in the analysis

The following list of concepts provides links to the Concepticon project [1] where Concepticon IDs are available.

Elicitation Gloss	Concepticon ID	Concepticon Gloss
ABOVE	1741	ABOVE
AFTER	1685	AFTER
AGOUTI	313	AGOUTI
ALL	98	ALL
ARM	1673	ARM
ARRIVE	1387	ARRIVE
ARROW	977	ARROW
AXE	677	AXE
BACK	1291	BACK
BANANA	868	BANANA
BARK OR SHELL	3865	BARK OR SHELL
BASKET	1539	BASKET
BAT	1793	BAT
BATHE	138	BATHE
BIG	1202	BIG
BIRD	937	BIRD
BITE	1403	BITE
BLACK	163	BLACK
BLOW (WITH MOUTH)	176	BLOW (WITH MOUTH)
BLUE	837	BLUE
BONE	1394	BONE
BOW	994	BOW
BOY	1366	BOY
BREAK (BREAKING)	3020	BREAK (BREAKING)
BREAST	1402	BREAST
BURN (SOMETHING)	141	BURN (SOMETHING)
BURY	1719	BURY
CAIMAN	2438	CAIMAN
CANOE	1970	CANOE
CLAW OR NAIL	2128	CLAW OR NAIL
CLOUD	1489	CLOUD
COLD	1287	COLD
COME	1446	COME
COME BACK	581	COME BACK
CONSUME (DRINK OR EAT)	2787	CONSUME (DRINK OR EAT)
COTTON	1850	COTTON
CRY	1839	CRY
CUT	1432	CUT
DANCE	1879	DANCE
DAUGHTER OF WOMAN		
DAUGHTER (OF MALE EGO)	3778	DAUGHTER (OF MALE EGO)
DAY (24 HOURS)	1260	DAY (24 HOURS)
DIE	1494	DIE
DIRTY	1230	DIRTY

DOG	2009	DOG
DO OR MAKE	2575	DO OR MAKE
DRY	1398	DRY
EARTH OR LAND	3603	EARTH OR LAND
EGG	744	EGG
ENTER	749	ENTER
EYE	1248	EYE
FACE	1560	FACE
FALL	1280	FALL
FAR	1406	FAR
FARM	201	FARM
FATHER	1217	FATHER
FAT (ORGANIC SUBSTANCE)	323	FAT (ORGANIC SUBSTANCE)
FEAR (BE AFRAID)	1419	FEAR (BE AFRAID)
FINGER	1303	FINGER
FINISH	1766	FINISH
FIRE	221	FIRE
FIREWOOD	10	FIREWOOD
FLESH OR MEAT	2615	FLESH OR MEAT
FLOUR	1594	FLOUR
FLOWER	239	FLOWER
FLY (INSECT)	1504	FLY (INSECT)
FLY (MOVE THROUGH AIR)	1441	FLY (MOVE THROUGH AIR)
FOOD	256	FOOD
FOOT	1301	FOOT
FROG	503	FROG
GIVE	1447	GIVE
GO	695	GO
GOOD	1035	GOOD
GO UP (ASCEND)	705	GO UP (ASCEND)
GOURD	411	GOURD
GRANDFATHER	1383	GRANDFATHER
GRASS	606	GRASS
GREEN	1425	GREEN
HAMMOCK	2019	HAMMOCK
HAND	1277	HAND
HAPPY	1495	HAPPY
HAWK	307	HAWK
HEAD	1256	HEAD
HEART	1223	HEART
HEN	1514	HEN
HIDE	2486	HIDE
HIT	1433	HIT
HOLE	1667	HOLE
HOUSE	1252	HOUSE
HUSBAND	1200	HUSBAND
I	1209	I
JAGUAR	1250	JAGUAR
KILL	1417	KILL
KNEE	1371	KNEE

KNIFE	1352	KNIFE
KNOW	3626	KNOW
LAKE	624	LAKE
LAUGH	1355	LAUGH
LEAF	628	LEAF
LEG	1297	LEG
LIVER	1224	LIVER
LONG	1203	LONG
LOOK	1819	LOOK
LOOK FOR	1468	LOOK FOR
LOUSE	1392	LOUSE
MAIZE	506	MAIZE
MAN	1554	MAN
MANIOC	927	MANIOC
MANY	1198	MANY
MEDICINE	1372	MEDICINE
MONKEY	1350	MONKEY
MOSQUITO	1509	MOSQUITO
MOTHER	1216	MOTHER
MOUTH	674	MOUTH
NECK	1333	NECK
NIGHT	1233	NIGHT
NOSE	1221	NOSE
ONE	1493	ONE
OTHER	197	OTHER
PAIN	1783	PAIN
PATH OR ROAD	2457	PATH OR ROAD
PERSON	683	PERSON
PIERCE	398	PIERCE
PISS	592	PISS
PLAY	1413	PLAY
PUT	998	PUT
RAIN (PRECIPITATION)	658	RAIN (PRECIPITATION)
RAT	1490	RAT
RED	156	RED
RIVER	666	RIVER
ROPE	1218	ROPE
RUN	1519	RUN
SALT	1274	SALT
SEE	1409	SEE
SEED	714	SEED
SIT DOWN	1649	SIT DOWN
SKY	1732	SKY
SMALL	1246	SMALL
SMOKE (EXHAUST)	778	SMOKE (EXHAUST)
SNAKE	730	SNAKE
SON OF WOMAN		
SON OF MAN	3779	SON (OF MALE EGO)
SPEAK	1623	SPEAK
SPIDER	843	SPIDER

STAND	1442	STAND
STAR	1430	STAR
STONE	857	STONE
STRONG	785	STRONG
SUN	1343	SUN
SWEET	717	SWEET
SWEET POTATO	159	SWEET POTATO
SWIM	1439	SWIM
TAIL	1220	TAIL
TAKE	1749	TAKE
TAPIR	312	TAPIR
TEACH	709	TEACH
TELL	1711	TELL
THING	738	THING
THORN	124	THORN
THREE	492	THREE
TICK	1527	TICK
TIE	1917	TIE
TIRED	1757	TIRED
TOBACCO	974	TOBACCO
TOMORROW	1329	TOMORROW
TONGUE	1205	TONGUE
TOOTH	1380	TOOTH
TREE	906	TREE
TWO	1498	TWO
VULTURE	1185	VULTURE
WALK	1443	WALK
WANT	1784	WANT
WASH	1453	WASH
WASP	1517	WASP
WE (EXCLUSIVE)	1130	WE (EXCLUSIVE)
WE (INCLUSIVE)	1131	WE (INCLUSIVE)
WET	1726	WET
WHITE	1335	WHITE
WIFE	1199	WIFE
WIND	960	WIND
WING	1257	WING
WOMAN	962	WOMAN
YELLOW	1424	YELLOW

Appendix B Neighbour-Net Delta Scores and Q-residuals

Taxon	Delta Score	Q-residual
Ache	0.394	0.0082955
Amondawa	0.3434	0.0052495
Anambe	0.42637	0.0062446
Apiaka	0.37362	0.0050591
Arawete	0.41031	0.0056964
Asuriní Tocantins	0.38347	0.0051849
Asuriní Xingu	0.37981	0.0060794
Ava Canoeiro	0.41997	0.0043306
Aweti	0.4134	0.0067146
Chiriguano	0.36944	0.005743
Guaja	0.42006	0.0060577
Guajajara	0.38565	0.0048662
Guaraní	0.36424	0.0054202
Guarayo	0.39375	0.0048682
Ka'apor	0.41211	0.0055927
Kaiowa	0.37258	0.0068028
Kamajura	0.41758	0.0058005
Kayabi	0.37631	0.0063377
Kokama	0.38402	0.0074942
Mawé	0.37891	0.0083243
Mbyá	0.36684	0.005574
Nheengatu	0.38522	0.0063162
Old Guaraní	0.36821	0.0055386
Omagua	0.37283	0.0058084
Parakana	0.38937	0.0050687
Parintintin	0.34267	0.0062729
Siriono	0.37968	0.0077449
Tapiete	0.38049	0.0053694
Tapirape	0.37465	0.0049149
Teko	0.38904	0.0054317
Tembe	0.40754	0.0056141
Tenharim	0.33317	0.0051776
Tupinamba	0.42605	0.0072714
Urueuwauwau	0.32172	0.0049375
Warazu	0.4261	0.0056621
Wayampi	0.40618	0.0050559
Xeta	0.37919	0.0061801
Yuki	0.37761	0.0091861
Zo'e	0.41124	0.0050511

Delta score = 0.3861
Q-residual score = 0.005957

Appendix C Heatmap of cognacy from Swadesh analysis

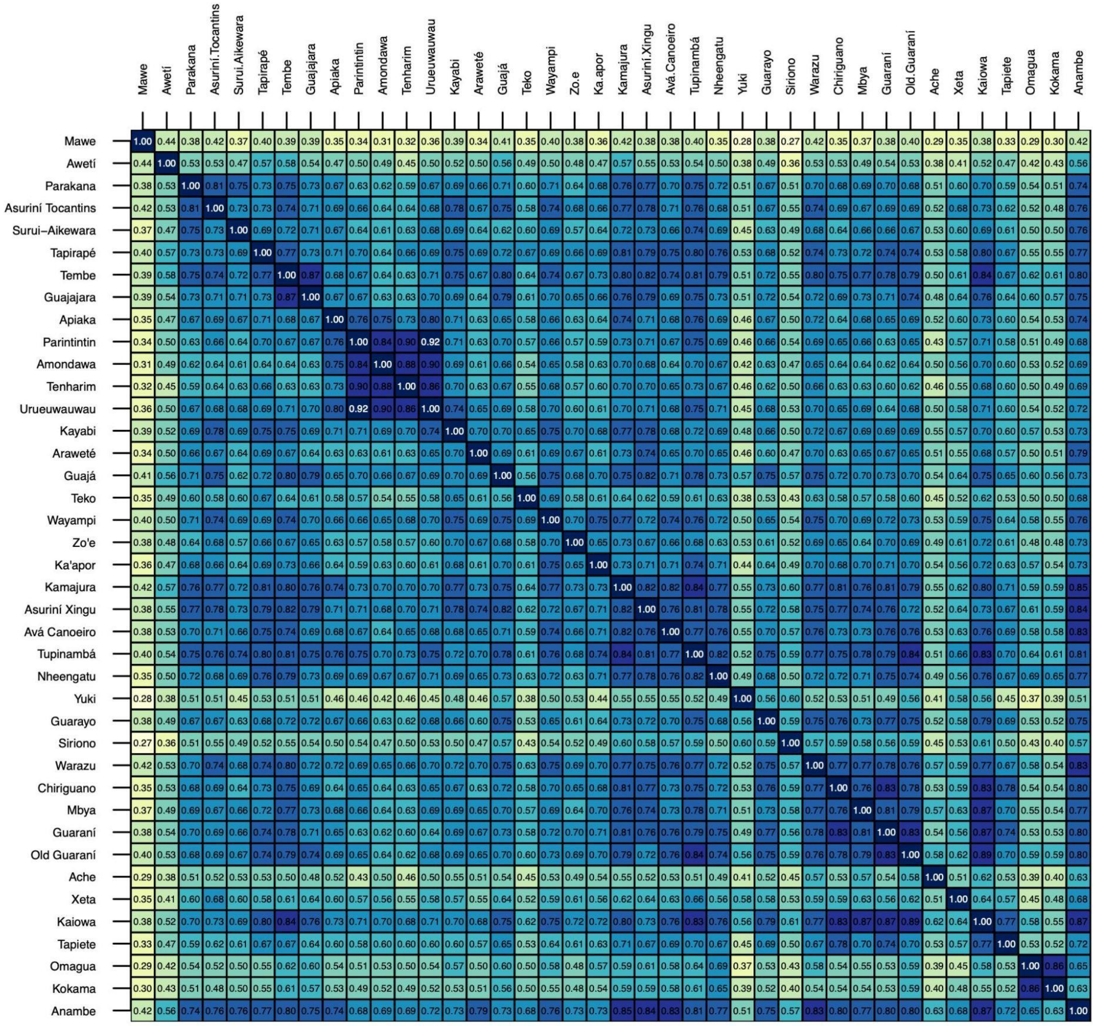

Fig 7. Cognacy for each language pair used in the Swadesh analysis (smaller set of concepts), ranging from 0 (full difference) to 1 (identity). The cognacy diversity [2] for all languages is 13.3%. If Mawé and Awetí are excluded, the value is 12.3%.

Appendix D Date calibrations and monophyletic constraints

Name	Languages	Date calibration
root	(all)	uniform (2500-5000)
awetitg	(all save for Mawé)	
tg	(all save for Mawé and Awetí)	
guaranian	Ache, Chiriguano, Guarani, Guarayo, Kaiowa, Mbya, Old_Guarani, Siriono, Tapiete, Warazu, Xeta, Yuki	
kawahiva	Amondawa, Apiaka, Kayabi, Parintintin, Tenharim, Urueuwauwau	
gtg	Guaja, Guajajara, Tembe	
zwt	Teko, Wayampi, Zoe	
tupi	Kokama, Nheengatu, Omagua, Tupinamba	
ok	Kokama, Omagua	

Appendix E Summary trees from the Bayesian analyses

E.1 Full cognate set, relaxed clock

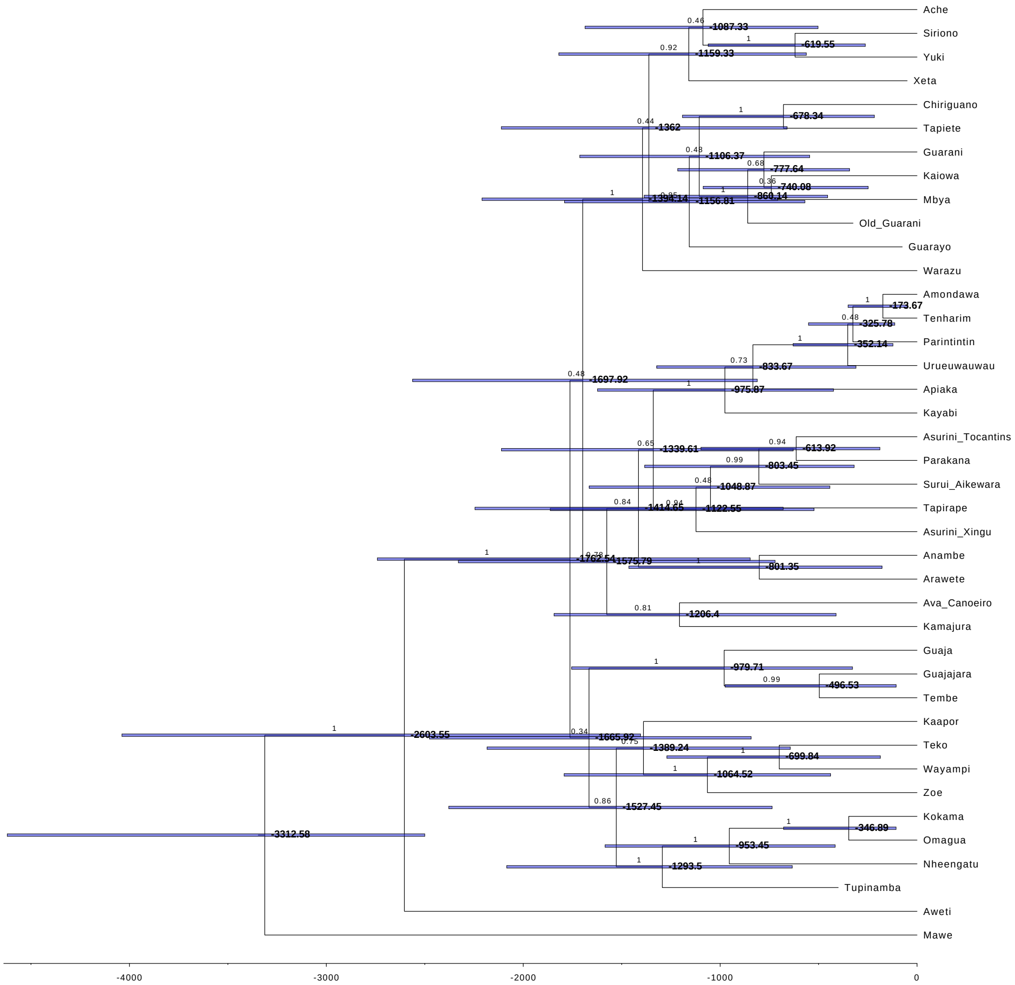

Fig 8. "full relaxed" MCC tree with split dates (node labels), 95% height (node bars), and posteriors (branch labels).

E.2 Full cognate set, strict clock

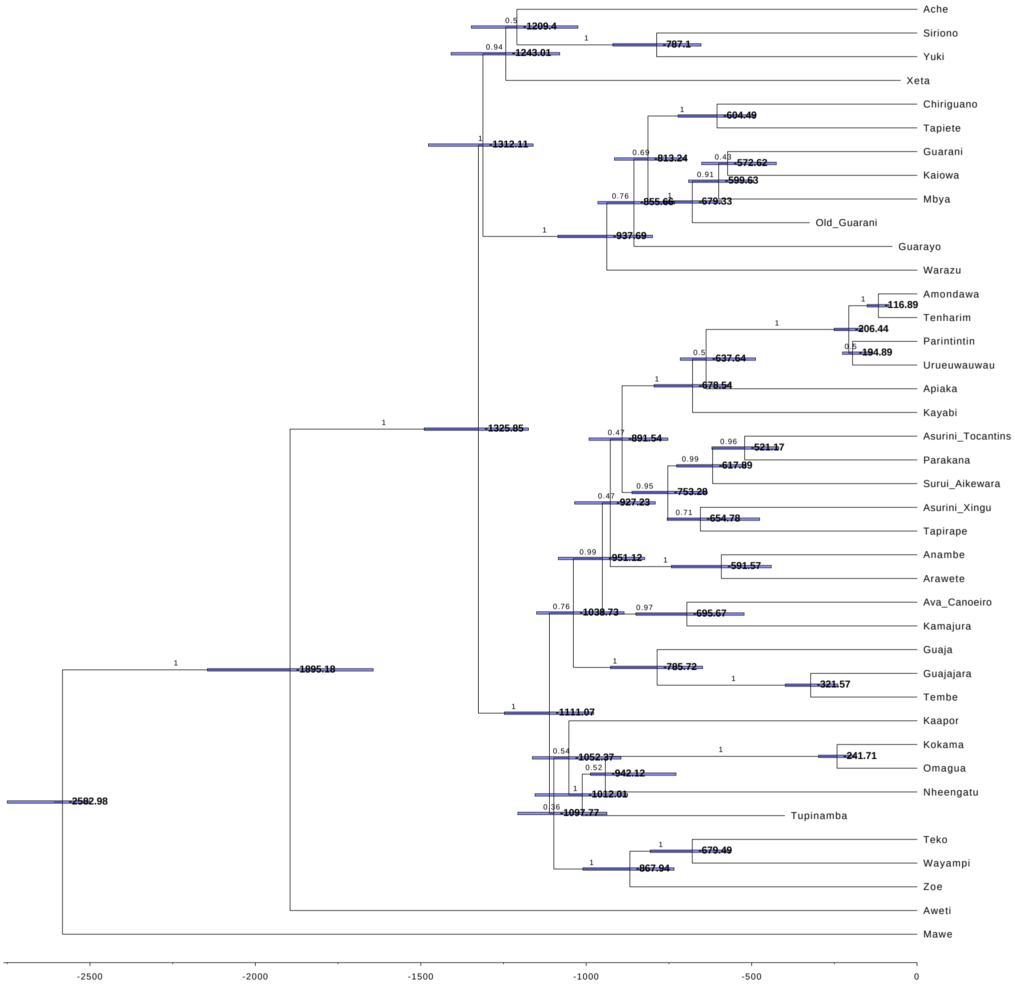

Fig 9. "full strict" MCC tree with split dates (node labels), 95% height (node bars), and posteriors (branch labels).

E.3 Swadesh cognate set, relaxed clock

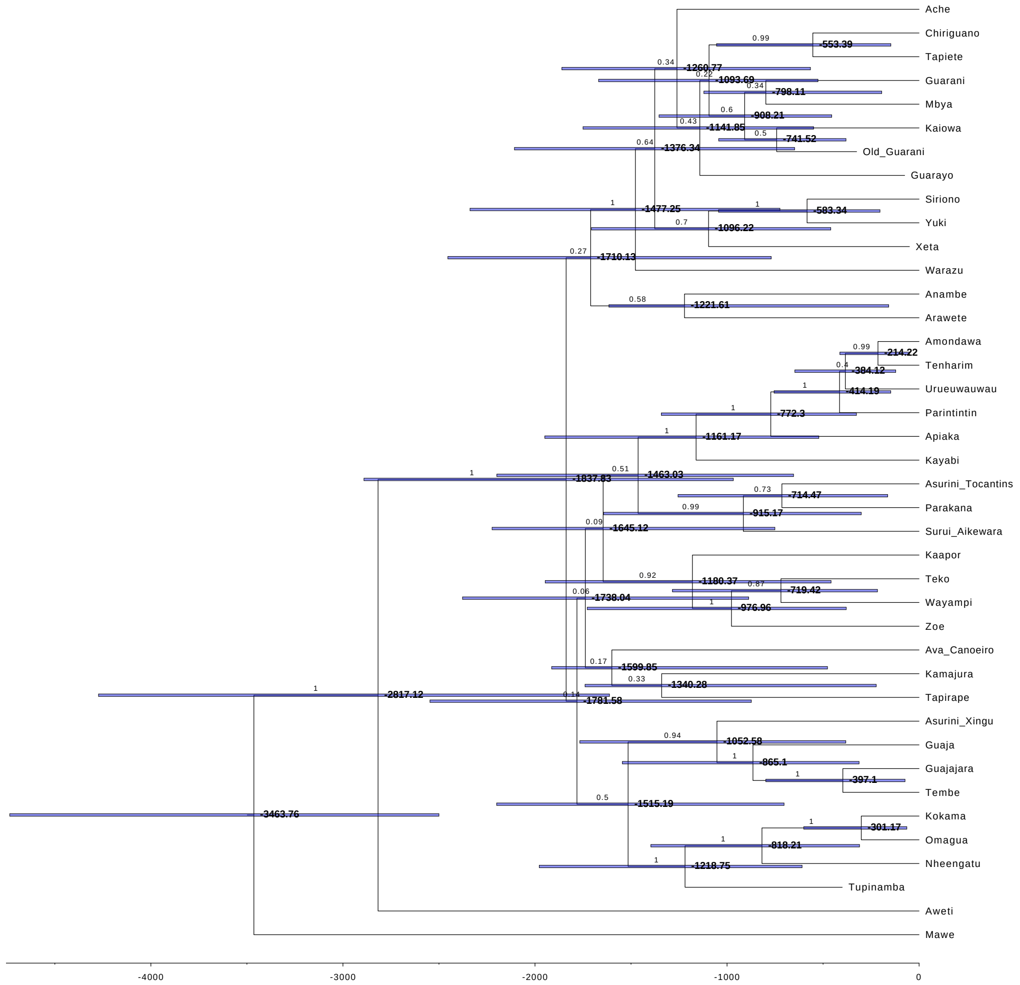

Fig 10. "swadesh relaxed" MCC tree with split dates (node labels), 95% height (node bars), and posteriors (branch labels).

E.4 Swadesh cognate set, strict clock

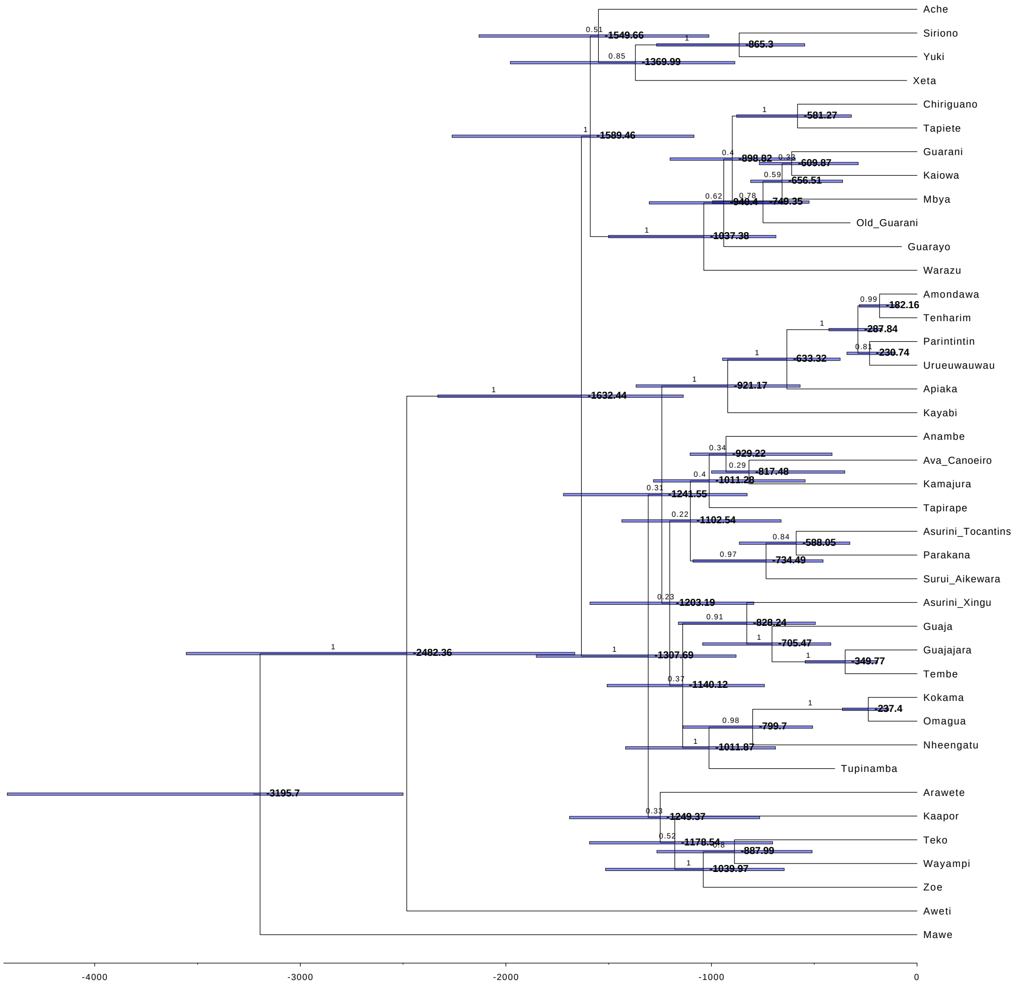

Fig 11. "swadesh strict" MCC tree with split dates (node labels), 95% height (node bars), and posteriors (branch labels).

Appendix F Densitree of "full" study

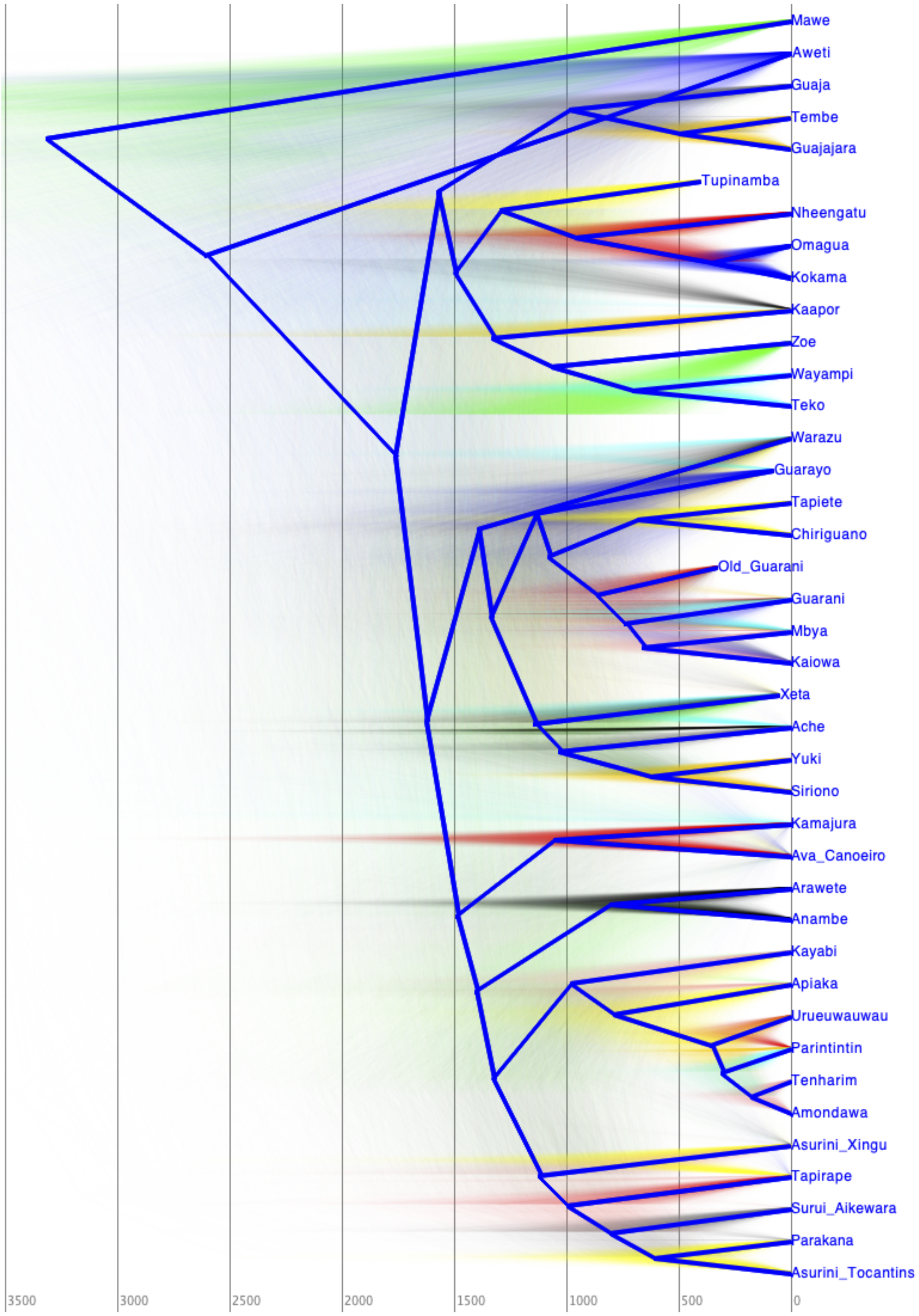

Fig 12. Density tree for the "main" study (birth-death, relaxed clock, large concept set).

Appendix G Full set of concepts used in the analyses

CONCEPT	COUNT	COVERAGE
above	40	0.9756
acaipalm	20	0.4878
achiote	31	0.7561
after	36	0.8780
agouti	37	0.9024
all	40	0.9756
anacondawaterboa	26	0.6341
ant	31	0.7561
anteater	34	0.8293
anus	19	0.4634
arm	39	0.9512
arrive	38	0.9268
arrow	40	0.9756
ash	35	0.8537
axe	39	0.9512
back	40	0.9756
badorevil	32	0.7805
bamboo	35	0.8537
banana	40	0.9756
barkorshell	37	0.9024
basket	39	0.9512
bat	38	0.9268
bathe	39	0.9512
bead	21	0.5122
bean	36	0.8780
beard	33	0.8049
beautiful	36	0.8780
bed	14	0.3415
bee	35	0.8537
belly	31	0.7561
bellybutton	36	0.8780
beloworunder	29	0.7073
bench	26	0.6341
big	41	1.0000
bird	39	0.9512
bite	41	1.0000
black	40	0.9756
blood	35	0.8537
blowwithmouth	40	0.9756
blue	38	0.9268
blueandyellowmacawaraararauna	18	0.4390
body	30	0.7317
bone	40	0.9756
bow	41	1.0000
boy	40	0.9756
branch	36	0.8780
brazilnutbertholletiaexcelsa	22	0.5366
breakbreaking	40	0.9756

breast	39	0.9512
breathe	35	0.8537
brother	16	0.3902
burning	35	0.8537
burnsomething	39	0.9512
bury	37	0.9024
butterfly	36	0.8780
buttocks	29	0.7073
caiman	38	0.9268
canoe	39	0.9512
capybara	27	0.6585
carry	23	0.5610
cashew	23	0.5610
catfish	31	0.7561
cebusmonkey	21	0.5122
centipede	22	0.5366
chicha	21	0.5122
chieftain	33	0.8049
child	37	0.9024
cicada	24	0.5854
clawornail	39	0.9512
clothes	23	0.5610
cloud	39	0.9512
coaticoatimundi	33	0.8049
cockroach	29	0.7073
cocoabean	20	0.4878
cold	41	1.0000
collaredpeccary	35	0.8537
comb	35	0.8537
come	40	0.9756
comeback	41	1.0000
consumedrinkoreat	41	1.0000
cooksomething	21	0.5122
cotton	38	0.9268
courtyard	18	0.4390
cricket	31	0.7561
cry	41	1.0000
curassow	30	0.7317
cure	34	0.8293
cut	41	1.0000
dance	40	0.9756
daughterofmaleego	41	1.0000
daughterofwoman	40	0.9756
day24hours	38	0.9268
deep	18	0.4390
deer	34	0.8293
die	41	1.0000
dig	30	0.7317
dirty	37	0.9024
dog	37	0.9024
door	34	0.8293
doormake	41	1.0000

dream	18	0.4390
dreamsomething	20	0.4878
dry	38	0.9268
dryup	36	0.8780
eagle	15	0.3659
ear	36	0.8780
earring	14	0.3415
earthorland	40	0.9756
egg	40	0.9756
electriceel	26	0.6341
enemy	24	0.5854
eye	41	1.0000
eyebrow	19	0.4634
face	37	0.9024
fall	41	1.0000
fanobject	22	0.5366
far	40	0.9756
farm	38	0.9268
fart	26	0.6341
father	41	1.0000
fatorganicsubstance	39	0.9512
fearbeafraid	41	1.0000
feather	35	0.8537
finger	38	0.9268
finish	38	0.9268
fire	41	1.0000
firefly	26	0.6341
firewood	37	0.9024
fishhook	28	0.6829
flat	36	0.8780
flea	26	0.6341
fleshormeat	40	0.9756
float	33	0.8049
flour	40	0.9756
flower	40	0.9756
flyinsect	39	0.9512
flymovethroughair	41	1.0000
food	39	0.9512
foot	41	1.0000
forehead	16	0.3902
forest	20	0.4878
forget	33	0.8049
four	35	0.8537
frighten	36	0.8780
frog	39	0.9512
fruit	37	0.9024
full	35	0.8537
gather	33	0.8049
genipa	26	0.6341
getlost	34	0.8293
girl	36	0.8780
give	39	0.9512

go	41	1.0000
good	41	1.0000
goupascend	38	0.9268
gourd	37	0.9024
grandfather	40	0.9756
grass	37	0.9024
green	38	0.9268
grind	18	0.4390
guan	28	0.6829
hair	41	1.0000
hammock	40	0.9756
hand	41	1.0000
happy	38	0.9268
hawk	37	0.9024
head	40	0.9756
heart	39	0.9512
hedgehog	24	0.5854
help	35	0.8537
hen	40	0.9756
here	37	0.9024
hide	37	0.9024
hit	41	1.0000
holdortake	17	0.4146
hole	38	0.9268
honey	37	0.9024
hopliasgenus	25	0.6098
hornanatomy	31	0.7561
house	41	1.0000
howlermonkey	32	0.7805
hummingbird	30	0.7317
hunt	34	0.8293
husband	39	0.9512
i	39	0.9512
ingagenus	18	0.4390
inside	27	0.6585
jaguar	41	1.0000
jump	30	0.7317
kill	41	1.0000
kingfisher	20	0.4878
knee	39	0.9512
knife	40	0.9756
know	40	0.9756
lake	38	0.9268
largebulletantsubfamilyparaponerinae	22	0.5366
larva	16	0.3902
laugh	41	1.0000
leaf	41	1.0000
learn	31	0.7561
leave	30	0.7317
leg	40	0.9756
liana	27	0.6585
liedown	24	0.5854

lightning	18	0.4390
lip	25	0.6098
liver	40	0.9756
lizard	34	0.8293
long	41	1.0000
look	40	0.9756
lookfor	39	0.9512
louse	40	0.9756
macaw	27	0.6585
maize	41	1.0000
man	41	1.0000
manioc	41	1.0000
many	40	0.9756
maternalgrandmother	36	0.8780
maternalunclemothersbrother	34	0.8293
medicine	39	0.9512
milk	26	0.6341
mirror	25	0.6098
moldgetrotten	14	0.3415
monkey	38	0.9268
morning	29	0.7073
mosquito	38	0.9268
mother	41	1.0000
motherinlawofman	29	0.7073
mountain	30	0.7317
moustache	30	0.7317
mouth	37	0.9024
mud	26	0.6341
mushfood	26	0.6341
name	35	0.8537
neck	39	0.9512
necklace	27	0.6585
nest	30	0.7317
new	35	0.8537
night	40	0.9756
nose	40	0.9756
now	31	0.7561
oar	25	0.6098
odorsmell	16	0.3902
old	31	0.7561
olderbrotherofman	32	0.7805
olderbrotherofwoman	28	0.6829
oldersisterofman	18	0.4390
oldersisterofwoman	17	0.4146
one	41	1.0000
open	25	0.6098
ornament	14	0.3415
other	40	0.9756
owl	32	0.7805
paca	36	0.8780
pacu	26	0.6341
pain	41	1.0000

paintsomething	34	0.8293
pan	35	0.8537
papaya	29	0.7073
parrot	38	0.9268
pathorroad	41	1.0000
peachpalm	15	0.3659
peanut	20	0.4878
penis	35	0.8537
pepper	28	0.6829
person	40	0.9756
pestle	23	0.5610
pierce	39	0.9512
pineapple	27	0.6585
piranha	29	0.7073
piss	36	0.8780
piumsimuliidae	25	0.6098
plantsomething	23	0.5610
play	41	1.0000
pumpkin	31	0.7561
put	37	0.9024
rainprecipitation	41	1.0000
rattle	16	0.3902
raw	23	0.5610
red	41	1.0000
redandgreenmacawarachloropterus	16	0.3902
remain	30	0.7317
remember	27	0.6585
resin	18	0.4390
ripe	35	0.8537
river	40	0.9756
roastsomething	28	0.6829
rope	38	0.9268
rotten	17	0.4146
round	35	0.8537
row	27	0.6585
run	40	0.9756
saliva	36	0.8780
salt	38	0.9268
sand	35	0.8537
say	37	0.9024
screamproduceacry	17	0.4146
see	40	0.9756
seed	38	0.9268
send	34	0.8293
shadow	27	0.6585
shamanfolkhealer	24	0.5854
sharpensomething	33	0.8049
shine	20	0.4878
shitdefecate	32	0.7805
short	30	0.7317
shoulder	22	0.5366
shrimp	14	0.3415

sick	36	0.8780
sieveorstrain	26	0.6341
sievetool	34	0.8293
sing	36	0.8780
sister	36	0.8780
sitdown	40	0.9756
sky	36	0.8780
sloth	25	0.6098
small	39	0.9512
smokeexhaust	39	0.9512
snail	28	0.6829
snake	40	0.9756
snore	28	0.6829
sonofman	41	1.0000
sonofwoman	41	1.0000
soundornoise	14	0.3415
sour	36	0.8780
speak	40	0.9756
spider	40	0.9756
spidermonkey	19	0.4634
sproutverb	15	0.3659
squirrel	23	0.5610
stand	41	1.0000
star	40	0.9756
stingray	34	0.8293
stinking	15	0.3659
stomach	27	0.6585
stone	41	1.0000
strong	37	0.9024
suck	32	0.7805
sun	40	0.9756
sweatsubstance	14	0.3415
sweep	34	0.8293
sweet	39	0.9512
sweetpotato	37	0.9024
swim	38	0.9268
tail	40	0.9756
take	37	0.9024
tapioca	18	0.4390
tapir	40	0.9756
tayraeirabarbara	16	0.3902
teach	38	0.9268
tell	39	0.9512
termite	29	0.7073
testicles	30	0.7317
that	34	0.8293
thick	29	0.7073
thing	40	0.9756
this	34	0.8293
thorn	40	0.9756
three	40	0.9756
throat	34	0.8293

throw	16	0.3902
thunder	36	0.8780
tick	38	0.9268
tie	38	0.9268
tinamou	27	0.6585
tired	40	0.9756
tobacco	39	0.9512
today	29	0.7073
tomorrow	40	0.9756
tongue	37	0.9024
tooth	37	0.9024
toucan	30	0.7317
tree	41	1.0000
tucumapalm	20	0.4878
turtle	37	0.9024
two	41	1.0000
ugly	36	0.8780
untie	35	0.8537
upperlegthigh	14	0.3415
vagina	31	0.7561
vein	34	0.8293
village	30	0.7317
vomit	35	0.8537
vulture	38	0.9268
wakeup	25	0.6098
walk	41	1.0000
want	40	0.9756
wash	39	0.9512
wasp	38	0.9268
water	41	1.0000
waterfall	26	0.6341
wax	17	0.4146
weave	23	0.5610
weexclusive	38	0.9268
weinclusive	41	1.0000
wet	38	0.9268
what	33	0.8049
white	41	1.0000
whitelippedpeccary	23	0.5610
who	36	0.8780
wife	40	0.9756
wildcat	23	0.5610
wind	39	0.9512
wing	39	0.9512
woman	41	1.0000
woodpecker	22	0.5366
wound	23	0.5610
wrong	28	0.6829
yam	25	0.6098
yellow	40	0.9756
yesterday	25	0.6098
youngerbrotherofman	30	0.7317

youngerbrotherofwoman	30	0.7317
youngersisterofman	15	0.3659
youngersisterofwoman	16	0.3902

Appendix H Full set of languages used in the analyses

DOCULECT	COUNT	COVERAGE
Ache	335	0.8072
Amondawa	306	0.7373
Anambe	203	0.4892
Apiaka	269	0.6482
Arawete	293	0.7060
Asurini_Tocantins	347	0.8361
Asurini_Xingu	261	0.6289
Ava_Canoeiro	326	0.7855
Aweti	382	0.9205
Chiriguano	371	0.8940
Guaja	331	0.7976
Guajajara	401	0.9663
Guarani	410	0.9880
Guarayo	370	0.8916
Ka'apor	396	0.9542
Kaiowa	212	0.5108
Kamajura	282	0.6795
Kayabi	262	0.6313
Kokama	340	0.8193
Mawe	367	0.8843
Mbya	350	0.8434
Nheengatu	378	0.9108
Old_Guarani	346	0.8337
Omagua	333	0.8024
Parakana	344	0.8289
Parintintin	398	0.9590
Siriono	389	0.9373
Surui_Aikewara	346	0.8337
Tapiete	320	0.7711
Tapirape	282	0.6795
Teko	397	0.9566
Tembe	388	0.9349
Tenharim	317	0.7639
Tupinamba	409	0.9855
Urueuwauwau	250	0.6024
Warazu	352	0.8482
Wayampi	410	0.9880
Xeta	252	0.6072
Yuki	265	0.6386
Zo'e	342	0.8241

Appendix I Set of concepts from our 'Swadesh' study used in the analyses

CONCEPT	COUNT	COVERAGE
all	39	0.9750
ash	34	0.8500
back	39	0.9750
belly	31	0.7750
big	40	1.0000
bird	38	0.9500
bite	40	1.0000
black	39	0.9750
blood	35	0.8750
bone	39	0.9750
breathe	34	0.8500
brother	16	0.4000
burning	34	0.8500
child	36	0.9000
clothes	22	0.5500
cloud	39	0.9750
cold	40	1.0000
come	39	0.9750
cooksomething	20	0.5000
count	10	0.2500
cry	40	1.0000
cut	40	1.0000
dance	39	0.9750
die	40	1.0000
dig	29	0.7250
dirty	37	0.9250
dog	36	0.9000
dry	37	0.9250
ear	35	0.8750
egg	39	0.9750
eye	40	1.0000
fall	40	1.0000
far	39	0.9750
father	40	1.0000
fearbeafraid	40	1.0000
feather	34	0.8500
fight	9	0.2250
fire	40	1.0000
float	32	0.8000
flower	40	1.0000
flymovethroughair	40	1.0000
foot	40	1.0000
forest	20	0.5000
four	34	0.8500
give	38	0.9500
good	40	1.0000

grass	36	0.9000
green	38	0.9500
hair	40	1.0000
hand	40	1.0000
head	39	0.9750
heart	38	0.9500
here	36	0.9000
hit	40	1.0000
hunt	33	0.8250
husband	39	0.9750
i	38	0.9500
kill	40	1.0000
lake	37	0.9250
laugh	40	1.0000
leaf	40	1.0000
leg	40	1.0000
liver	39	0.9750
long	40	1.0000
louse	40	1.0000
man	40	1.0000
many	39	0.9750
mother	40	1.0000
mountain	30	0.7500
mouth	36	0.9000
name	35	0.8750
neck	38	0.9500
new	35	0.8750
night	40	1.0000
nose	39	0.9750
old	31	0.7750
one	40	1.0000
other	39	0.9750
person	39	0.9750
play	40	1.0000
pull	9	0.2250
push	10	0.2500
rainprecipitation	40	1.0000
red	40	1.0000
river	39	0.9750
rope	37	0.9250
rotten	17	0.4250
salt	37	0.9250
sand	35	0.8750
see	39	0.9750
seed	38	0.9500
short	30	0.7500
sing	36	0.9000
sister	35	0.8750
sky	36	0.9000
small	38	0.9500
smokeexhaust	38	0.9500
snake	39	0.9750

speak	39	0.9750
stand	40	1.0000
star	39	0.9750
stone	40	1.0000
straight	9	0.2250
suck	31	0.7750
sun	39	0.9750
swell	10	0.2500
swim	37	0.9250
tail	40	1.0000
that	33	0.8250
thick	28	0.7000
think	9	0.2250
this	33	0.8250
three	39	0.9750
throw	16	0.4000
tie	37	0.9250
tongue	37	0.9250
tooth	37	0.9250
tree	40	1.0000
two	40	1.0000
vomit	35	0.8750
walk	40	1.0000
wash	38	0.9500
water	40	1.0000
wet	38	0.9500
what	33	0.8250
white	40	1.0000
who	36	0.9000
wife	39	0.9750
wind	38	0.9500
wing	39	0.9750
woman	40	1.0000
yellow	39	0.9750

Appendix J Set of languages from our 'Swadesh' study used in the analyses

DOCULECT	COUNT	COVERAGE
Ache	129	0.9773
Amondawa	112	0.8485
Anambe	92	0.6970
Apiaka	108	0.8182
Arawete	109	0.8258
Asurini_Tocantins	117	0.8864
Asurini_Xingu	102	0.7727
Ava_Canoeiro	120	0.9091
Aweti	123	0.9318
Chiriguano	132	1.0000
Guaja	110	0.8333
Guajajara	129	0.9773
Guarani	131	0.9924
Guarayo	123	0.9318
Ka'apor	119	0.9015
Kaiowa	87	0.6591
Kamajura	109	0.8258
Kayabi	101	0.7652
Kokama	116	0.8788
Mawe	123	0.9318
Mbya	118	0.8939
Nheengatu	121	0.9167
Old_Guarani	116	0.8788
Omagua	122	0.9242
Parakana	117	0.8864
Parintintin	123	0.9318
Siriono	131	0.9924
Surui_Aikewara	118	0.8939
Tapiete	118	0.8939
Tapirape	112	0.8485
Teko	130	0.9848
Tembe	130	0.9848
Tenharim	111	0.8409
Tupinamba	128	0.9697
Urueuwauwau	104	0.7879
Warazu	119	0.9015
Wayampi	128	0.9697
Xeta	91	0.6894
Yuki	115	0.8712
Zo'e	113	0.8561

Appendix K Corrected TIGER scores for concepts

TIGER scores for all concepts in the "main" and "swadesh" datasets, following the implementation by [3].

The "main" dataset has a mean TIGER score of 0.14 (±0.14), while the "swadesh" one has a mean TIGER score of 0.14 (±0.17).

CONCEPT	MAIN	SWADESH
above	0.1013	-
acaipalm	0.0297	-
achiote	0.1981	-
after	0.0807	-
agouti	0.3566	-
all	0.2355	0.2228
anacondawaterboa	0.0263	-
ant	0.1854	-
anteater	0.1475	-
anus	0.0442	-
arm	0.4965	-
arrive	0.0522	-
arrow	0.2770	-
ash	0.1313	0.1478
axe	0.1886	-
back	0.1761	0.1788
badorevil	0.0183	-
bamboo	0.1831	-
banana	0.0995	-
barkorshell	0.0569	-
basket	0.0323	-
bat	0.1518	-
bathe	0.3452	-
bead	0.0294	-
bean	0.2106	-
beard	0.1151	-
beautiful	0.0694	-
bed	0.0038	-
bee	0.0220	-
belly	0.0294	0.0539
bellybutton	0.1447	-
beloworunder	0.2442	-
bench	0.0860	-
big	0.0782	0.0816
bird	0.3455	0.3815
bite	0.4667	0.4288
black	0.2812	0.2534
blood	0.0935	0.1107
blowwithmouth	0.3532	-
blue	0.1130	-
blueandyellowmacawaraararauna	0.0144	-
body	0.0684	-
bone	0.6084	0.5860
bow	0.4362	-

boy	0.0745	-
branch	0.3559	-
brazilnutbertholletiaexcelsa	0.0214	-
breakbreaking	0.0593	-
breast	0.3233	-
breathe	0.1029	0.0989
brother	0.0225	0.0273
burning	0.1161	0.1107
burnsomething	0.1580	-
bury	0.2757	-
butterfly	0.1298	-
buttocks	0.0345	-
caiman	0.2403	-
canoe	0.1000	-
capybara	0.0636	-
carry	0.0309	-
cashew	0.1099	-
catfish	0.0851	-
cebusmonkey	0.0610	-
centipede	0.0332	-
chicha	0.0370	-
chieftain	0.0140	-
child	0.0073	0.0124
cicada	0.1037	-
clawornail	0.2030	-
clothes	0.0110	0.0164
cloud	0.0757	0.0753
coaticoatimundi	0.1744	-
cockroach	0.1164	-
cocoabean	0.0346	-
cold	0.1687	0.1924
collaredpeccary	0.1223	-
comb	0.3172	-
come	0.0517	0.0778
comeback	0.2173	-
consumedrinkoreat	0.1957	-
cooksomething	0.0332	0.0632
cotton	0.3379	-
count	-	0.0377
courtyard	0.0341	-
cricket	0.1485	-
cry	0.7352	0.7486
curassow	0.2387	-
cure	0.0570	-
cut	0.1378	0.1662
dance	0.0404	0.0436
daughterofmaleego	0.2218	-
daughterofwoman	0.1195	-
day24hours	0.1116	-
deep	0.0322	-
deer	0.0378	-
die	0.5139	0.5430

dig	0.0398	0.0312
dirty	0.0606	0.0718
dog	0.1120	0.1138
door	0.0451	-
doormake	0.0938	-
dream	0.0076	-
dreamsomething	0.0366	-
dry	0.0452	0.0520
dryup	0.0376	-
eagle	0.0189	-
ear	0.3083	0.2668
earring	0.0432	-
earthorland	0.4242	-
egg	0.5759	0.5055
electriceel	0.0305	-
enemy	0.0090	-
eye	0.4940	0.4838
eyebrow	0.0284	-
face	0.3035	-
fall	0.2267	0.2639
fanobject	0.1116	-
far	0.0287	0.0394
farm	0.2993	-
fart	0.1903	-
father	0.2065	0.2436
fatorganicsubstance	0.1193	-
fearbeafraid	0.4758	0.4982
feather	0.0366	0.0602
fight	-	0.0000
finger	0.0310	-
finish	0.1351	-
fire	0.5654	0.6117
firefly	0.0194	-
firewood	0.0770	-
fishhook	0.0957	-
flat	0.1296	-
flea	0.0296	-
fleshormeat	0.1806	-
float	0.1232	0.1603
flour	0.1209	-
flower	0.4088	0.3833
flyinsect	0.3556	-
flymovethroughair	0.7352	0.7486
food	0.1177	-
foot	0.6606	0.5968
forehead	0.0382	-
forest	0.0026	0.0078
forget	0.1094	-
four	0.0682	0.0792
frighten	0.1010	-
frog	0.1368	-
fruit	0.0552	-

full	0.0727	-
gather	0.0782	-
genipa	0.1684	-
getlost	0.1074	-
girl	0.0433	-
give	0.1110	0.1160
go	0.7352	-
good	0.2534	0.2587
goupascend	0.2063	-
gourd	0.0742	-
grandfather	0.3384	-
grass	0.0632	0.0659
green	0.0893	0.1103
grind	0.0064	-
guan	0.0768	-
hair	0.1183	0.1246
hammock	0.0931	-
hand	0.2389	0.2519
happy	0.2546	-
hawk	0.0545	-
head	0.3374	0.2889
heart	0.1370	0.1583
hedgehog	0.0951	-
help	0.1228	-
hen	0.0746	-
here	0.1161	0.1306
hide	0.4193	-
hit	0.1605	0.1437
holdortake	0.0230	-
hole	0.3011	-
honey	0.1957	-
hopliasgenus	0.0579	-
hornanatomy	0.0546	-
house	0.0620	-
howlermonkey	0.0494	-
hummingbird	0.0708	-
hunt	0.0201	0.0199
husband	0.1115	0.1320
i	0.3217	0.3138
ingagenus	0.0409	-
inside	0.1039	-
jaguar	0.2484	-
jump	0.1148	-
kill	0.4246	0.4020
kingfisher	0.0345	-
knee	0.0932	-
knife	0.2321	-
know	0.6581	-
lake	0.1648	0.1531
largebulletantsubfamilyparaponerinae	0.0828	-
larva	0.0109	-
laugh	0.1968	0.2017

leaf	0.0311	0.0428
learn	0.1337	-
leave	0.1035	-
leg	0.2515	0.2590
liana	0.0509	-
liedown	0.0036	-
lightning	0.0479	-
lip	0.1625	-
liver	0.4601	0.3821
lizard	0.1118	-
long	0.5190	0.4837
look	0.2129	-
lookfor	0.2926	-
louse	0.6955	0.6743
macaw	0.0756	-
maize	0.5428	-
man	0.2002	0.2526
manioc	0.3942	-
many	0.1807	0.2044
maternalgrandmother	0.2507	-
maternalunclemothersbrother	0.0771	-
medicine	0.3846	-
milk	0.0834	-
mirror	0.0177	-
moldgetrotten	0.0325	-
monkey	0.2696	-
morning	0.0483	-
mosquito	0.2218	-
mother	0.0701	0.0920
motherinlawofman	0.0326	-
mountain	0.2152	0.2341
moustache	0.1039	-
mouth	0.3562	0.3706
mud	0.0437	-
mushfood	0.0118	-
name	0.2427	0.2352
neck	0.2214	0.2422
necklace	0.0906	-
nest	0.1215	-
new	0.1547	0.1654
nose	0.0647	0.0916
now	0.0225	-
oar	0.1592	-
odorsmell	0.0134	-
old	0.0393	0.0519
olderbrotherofman	0.1296	-
olderbrotherofwoman	0.0273	-
oldersisterofman	0.0254	-
oldersisterofwoman	0.0100	-
one	0.0595	0.0571
open	0.0110	-
ornament	0.0031	-

other	0.2053	0.2236
owl	0.0675	-
paca	0.1128	-
pacu	0.0935	-
pain	0.2456	-
paintsomething	0.0136	-
pan	0.0566	-
papaya	0.0314	-
parrot	0.1037	-
pathorroad	0.5996	-
peachpalm	0.0310	-
peanut	0.0716	-
penis	0.0858	-
pepper	0.0490	-
person	0.0960	0.1112
pestle	0.0143	-
pierce	0.0632	-
pineapple	0.0787	-
piranha	0.1206	-
piss	0.3825	-
piumsimuliidae	0.0216	-
plantsomething	0.0896	-
play	0.0978	0.0989
pull	-	0.0522
pumpkin	0.0449	-
push	-	0.0078
put	0.0793	-
rainprecipitation	0.3702	0.3024
rattle	0.0318	-
raw	0.0395	-
red	0.2411	0.2454
redandgreenmacawarachloropterus	0.0083	-
remain	0.0792	-
remember	0.0367	-
resin	0.0129	-
ripe	0.0317	-
river	0.0548	0.0537
roastsomething	0.0377	-
rope	0.0726	0.0686
rotten	0.0025	0.0098
round	0.2078	-
row	0.1077	-
run	0.3188	-
saliva	0.2819	-
salt	0.0778	0.0788
sand	0.3806	0.4100
say	0.0907	-
screamproduceacry	0.0164	-
see	0.1100	0.1432
seed	0.1883	0.1907
send	0.2295	-
shadow	0.0657	-

shamanfolkhealer	0.0895	-
sharpensomething	0.1187	-
shine	0.0125	-
shitdefecate	0.0759	-
short	0.0172	0.0244
shoulder	0.1277	-
shrimp	0.0643	-
sick	0.0804	-
sieveorstrain	0.0968	-
sievetool	0.0849	-
sing	0.0227	0.0363
sister	0.0395	0.0466
sitdown	0.4498	-
sky	0.1202	0.0884
sloth	0.0970	-
small	0.0422	0.0473
smokeexhaust	0.2471	0.2532
snail	0.0183	-
snake	0.4867	0.4478
snore	0.1257	-
sonofman	0.1853	-
sonofwoman	0.2236	-
soundornoise	0.0241	-
sour	0.0609	-
speak	0.2055	0.1734
spider	0.3051	-
spidermonkey	0.1153	-
sproutverb	0.0022	-
squirrel	0.0552	-
stand	0.1679	0.1938
star	0.2638	0.2619
stingray	0.1240	-
stinking	0.0139	-
stomach	0.0198	-
stone	0.3047	0.3376
straight	-	0.0168
strong	0.0734	-
suck	0.0770	0.0839
sun	0.3590	0.3434
sweatsubstance	0.0071	-
sweep	0.0729	-
sweet	0.1483	-
sweetpotato	0.2351	-
swell	-	0.0354
swim	0.1631	0.2105
tail	0.2531	0.2538
take	0.1929	-
tapioca	0.0333	-
tapir	0.1332	-
tayraeirabarbara	0.0436	-
teach	0.2202	-
tell	0.0693	-

termite	0.1611	-
testicles	0.2159	-
that	0.0272	0.0316
thick	0.0313	0.0367
thing	0.2595	-
think	-	0.0310
this	0.0627	0.0916
thorn	0.2814	-
three	0.0593	0.0602
throat	0.0485	-
throw	0.0222	0.0394
thunder	0.0438	-
tick	0.2857	-
tie	0.1016	0.1200
tinamou	0.1790	-
tired	0.0506	-
tobacco	0.1023	-
today	0.0096	-
tomorrow	0.0337	-
tongue	0.1807	0.1969
tooth	0.0813	0.0952
toucan	0.1708	-
tree	0.1807	0.2225
tucumapalm	0.0292	-
turtle	0.0726	-
two	0.2178	0.1955
ugly	0.0103	-
untie	0.0668	-
upperlegthigh	0.0091	-
vagina	0.0062	-
vein	0.0459	-
village	0.0583	-
vomit	0.0377	0.0474
vulture	0.3523	-
wakeup	0.0514	-
walk	0.5654	0.6117
want	0.1945	-
wash	0.0426	0.0446
wasp	0.3624	-
water	0.4690	0.4354
waterfall	0.0451	-
wax	0.0276	-
weave	0.0224	-
weexclusive	0.3615	-
weinclusive	0.0875	-
wet	0.0720	0.0744
what	0.1348	0.1801
white	0.2967	0.3312
whitelippedpeccary	0.0423	-
who	0.1446	0.1776
wife	0.1055	0.1273
wildcat	0.0605	-

wind	0.5112	0.4664
wing	0.1253	0.1208
woman	0.2611	0.2730
woodpecker	0.0316	-
wound	0.0233	-
wrong	0.0717	-
yam	0.0974	-
yellow	0.1147	0.1602
yesterday	0.0828	-
youngerbrotherofman	0.0544	-
youngerbrotherofwoman	0.0396	-
youngersisterofman	0.0140	-
youngersisterofwoman	0.0014	-

Appendix L Nested Samples output

MODEL	MARGINAL LIKELIHOOD	STANDARD DEVIATION
full.relaxed	-29725	7.49
full.strict	-21065	11.98
swadesh.relaxed	-12971	5.39
swadesh.strict	-13348	6.02

References

1. List JM, Rzymski C, Greenhill S, Schweikhard N, Pianykh K, Tjuka A, et al.. Concepticon 2.5.0; 2021. Available from: https://concepticon.clld.org/.

2. List JM, Greenhill SJ, Gray RD. The potential of automatic word comparison for historical linguistics. PLOS One. 2017;12(1):e0170046.

3. List JM. Correcting a bias in TIGER rates resulting from high amounts of invariant and singleton cognate sets. Journal of Language Evolution. 2022;doi:10.1093/jole/lzab007.
