# Supplementary material for: Lexical phylogenetics of the Tupí-Guaraní family: Language, archaeology, and the problem of chronology
Source: PLoS One. 2023 Jun 15;18(6):e0272226. doi: 10.1371/journal.pone.0272226 (PMC10270611; doi:10.1371/journal.pone.0272226)
Supplement: Attachment — Submitted filename: Evaluation of the manuscript by Gerardi et al. 2021..docx [file pone.0272226.s002.docx]

EVALUATION

The manuscript “Lexical Phylogenetics of the Tupí-Guaraní Family: Language, Archaeology, and the Problem of Chronology” written by Gerardi et al. discusses much debated problem of Tupí-Guaraní expansion.

The authors correctly take in account current early radiocarbon dates related to ceramics associated to Tupí-Guaraní language groups, and they also make correct reference to some historical and ethnographic information of these groups. In general, the authors utilize quite comprehensive linguistic database of cognate data, that is, importantly, open for everyone. In their actual analysis they use Bayesian phylogenic methods in order to build Tupí-Guaraní expansion model with corresponding chronological estimations. Finally, the results are compared and contrasted to archaeological data that is summarized at the beginning of the study.

I am not specialist of lexical phylogenetics, but the supporting information, figures and tables help considerable the reading, even though, I must admit, the quality of Figures 1 and 2 are poor. Also specific uncertainties and the inclusion of special cases such as Omagua and Kokama are well explained - making the analysis convincing. The authors propose, in the text, a major split at ca. 950 BP dividing Tupí-Guaraní languages in two major groups, (1.) the southern group in southern Brazil, Paraguay, Bolivia and Argentina, and (2) all other Amazonian TG languages and Tupinambá divided into four clades (pages 9-10). Nevertheless, in the Abstract the split between Southern and Northern varieties is dated as 1100 BP.

In general, the authors announce “the importance of developing an interdisciplinary unified model incorporating data from both disciplines (archaeology and linguistics).” Undoubtedly this is a good objective even though not easy to achieve. First of all, one should remember that only rarely can a one-to-one relationship between the tangible and intangible evidence be demonstrated. Linguistically or historically recorded social, religious, political or linguistic changes do not immediately affect all material culture which can be detected archaeologically, or vice versa; a rapid change in material culture does not necessarily imply a simultaneous reorganization of social, religious, political or linguistic life (e.g. Braudel 1980:25–54, 64–82). In numerous cases, the lack of correlation between archaeological and intangible evidence has been documented (e.g. Pärssinen & Siiriäinen 1997; Marsh et al 2017). Also in Mexico, Michael E. Smith (1987) analyzed ethnohistorical and archaeological records of the Aztec expansion and concluded that the supposed artefactual markers of conquest spread to some provincial regions before the actual incorporation of these regions into the Aztec state. In a similar vein, Thomas Charlton (1981) has demonstrated that shifts from one period to another do not necessarily occur simultaneously in Mexican historical and archaeological sequences. Hence, before archaeologists and linguistics have agreed the exact meaning of concepts they are using, records of different disciplines should be analyzed separately to yield independent conclusions before correlation is attempted. As Smith (1987) proposed: “When the two records [of different disciplines] are compared, one should not confuse any resulting composite models with the independent primary data sets.” In other words, one should take in account the comparability of the data sets (so called Galton´s problem), and to be careful when using analogically models of other discipline. As Max Black (1962) once said: “Any would-be scientific use of an analogue model demands independent confirmation. Analogue models furnish plausible hypotheses, not proofs.”

After pointing these multi- and interdisciplinary problems, I must congratulate the authors of current manuscript to separate their linguistics results from early C14-dates obtained by archaeologists during their excavations. It is not surprising that finally the authors point out “the difficulties in reconciling archaeological and linguistic data.” In fact, even though the conservative behavior of historical Tupí-Guaraní in their material and economic cultures (p.12) can be accepted *a posteriori*, it does not mean that we may freely apply this statement analogically, *a priori*, to the situation around 2000 BP. Recently Pärssinen (2021) has published evidence from the Brazilian Acre, that in the Upper Purus corrugated, finger-nail and some polychrome decoration in grog tempered ceramics, often associated to Tupí-Guaraní styles, existed already 2000 calBP mixed with ceramics associated with so called Arawakan styles - all pointing to some kind of multicultural entity that built geometrically patterned ceremonial centers called “geoglyphs” in the region. Interestingly, also Pärssinen criticizes too keen association of material remains to linguistic groups when referring to pan-Amazonian traditions. Similarly with Anna Roosevelt and Denise Schaan he prefers to return to more neutral term such as Polychrome Horizon, and in fact, divides the Horizon into two phases, the Early (ca. 300 BC – 300/500 AD) and the Late Polychrome Horizon (ca. 900 – 1550 AD). Nevertheless, even though both Horizons were multicultural, he admits that that the first Horizon may partially be related to Arawakan expansion and the second one to Tupí-Guaraní expansion (see also, e.g. Almeida & Moraes 2016; Almeida & Neves 2014). Interestingly, this last period based on various C14 dating, seems to correlate quite well with the main results of the manuscript of Gerardi et al.

To conclude, I consider the manuscript of Gerardi et al. well written and its´ results to be an important contribution for multidisciplinary Tupí-Guaraní Studies. It also contains points of general interest, and in general, it satisfied all the publication criteria established by PLOS ONE. Hence, I recommend its´ publication with minor modifications.

References cited:

Almeida, F.O. & Moraes, C.P. 2016. A cerâmica polícroma do rio Madeira, in *Cerâmicas arqueológicas da Amazônia: Rumo a uma nova síntese*. Edited by C. Barreto, H. P. Lima, & C. Jaimes Betancourt, pp. 402-413. Belém: Iphan, Museu Paraense Emílio Goeldi.

Almeida, F.O. & Neves, E.G. 2014. The polychrome tradition at the Upper Madeira river, in *Antes de Orellana. Actas del 3er Encuentro Internacional de Arqueología Amazónica*. Edited by S. Rostain, v. 37, pp. 175-182. Quito: Instituto Francés de Estudios Andinos.

Black, M.1962. *Models and Metaphors: Studies in Language and Philosophy*. Ithaca, New York: Cornell University Press.

Braudel, F. 1980. *On History*. Translated by Sarah Matthews. London: Weidenfeld and Nicolson.

Charlton, T. H. 1981. Archaeology, Ethnohistory, and Ethnology: Interpretive Interfaces. *Advances in Archaeological Method and Theory* 4:129–176.

Marsh, Erik J., Ray Kidd, Dennis Ogburn & Victor Durán (2017). Dating the Expansion of the Inca Empire: Bayesian Models from Ecuador and Argentina. *Radiocarbon* 59:1, 117–140.

Pärssinen, M. 2021. Tequinho Geoglyph Site and Early Polychrome Horizon 300 BC - AD 300/500 in the Brazilian State of Acre. Amazônica: Revista de Antropologia, 13/2021(1), 177-220. DOI: <http://dx.doi.org/10.18542/amazonica.v13i1.9095>

Pärssinen, M. & A. Siiriäinen. 1997. Inka-style ceramics and their chronological relationship to the Inka expansion in the southern Lake Titicaca area (Bolivia). *Latin American Antiquity,* 8(3): 255–271.

Smith, M.E. 1987. The expansion of the Aztec empire: A case study in the correlation of diachronic archaeological and ethnohistorical data. *American Antiquity* 52(1): 37 – 54.
